# Frequency of Gastrointestinal Parasites, Anemia, and Nutritional Status among Children from Different Geographical Regions of Bolivia

**DOI:** 10.1155/2023/5020490

**Published:** 2023-12-09

**Authors:** Ceilan Apaza, Washington Cuna, Froilán Brañez, Roberto Passera, Celeste Rodriguez

**Affiliations:** ^1^Hospital Municipal de Chulumani, Chulumani, La Paz, Bolivia; ^2^Unidad de Inmunología Parasitaria, Facultad de Medicina, Universidad Mayor de San Andrés, La Paz, Bolivia; ^3^Unidad de Laboratorio Clínico, Hospital de Caranavi, Caranavi, La Paz, Bolivia; ^4^University of Turin, Department of Medical Science, Division of Nuclear Medicine, Corso AM Digliotti 14, Turin 10126, Italy

## Abstract

The study aimed to measure the frequency of occurrence of infections with helminths, protozoa, and risk factors of undernutrition and anemia among schoolchildren from the Bolivian highland (altiplano) and lowland (subtropical) rural regions, with a high frequency of gastrointestinal parasite infections. Cross-sectional data were collected from 790 children, 5–13 years old. Microscopic examination of stool using the Ritchie technique, hemoglobin testing using the HemoCue analyzer, and anthropometric measurements were performed. Over 60% and 20% of children were infected with protozoa and helminth parasites, respectively. Infections caused by pathogenic *Hymenolepis nana* (15.7–5.2%), *Ascaris lumbricoides* (41.9–28.5%), *Giardia lamblia* (30.1–11.2%), *Entamoeba histolytica* (5.7–0.7%), and nonpathogenic *Entamoeba coli* (48.9–16%), *Blastocystis hominis* (40.2–28.5%), *Iodamoeba butschli* (16.1–2.5%), *Chilomastix mesnili* (19.2–7.3%), and *Entamoeba histolytica/dispar* (7.4–5.5%) parasites, were more prevalent in the highlands than the lowlands. Single parasitic infections were more prevalent in the lowlands; polyparasitism of light or heavy intensity predominated in the highlands. A strongly increased risk of anemia and a low prevalence of wasting were determined in children in the highlands. A higher risk for stunting was associated with children of older age, and a low burden of intestinal helminths would prevent wasting in children of highlands. Infections with *A. lumbricoides* and *G. lamblia* pathogens in older children were not significant covariates for stunting. Environmental, nutritional, and parasitic factors may predispose to anemia in the highlands. A nutritional intervention and parasite control effort will substantially improve children´s health in the highlands.

## 1. Introduction

Parasitic infections by helminths and/or protozoa are among the oldest pervading classes of infections globally, affecting more than 1 billion people in marginalized and poor communities [1–3]. Infections with helminths including *Ascaris lumbricoides, Hymenolepis nana*, and protozoa (e.g., *Entamoeba histolytica* and *Giardia lamblia*), are usually observed in people from developing countries living in conditions of poverty where clean water, proper housing, and sewage systems are not available [4, 5]. School-age children carry the heaviest burden of morbidity associated with infectious diseases in developing countries. Although mortality rates associated with parasitic diseases of the gastrointestinal tract are relatively low compared to other infectious diseases, persistent infections result in malnutrition and anemia, leading to downstream effects of undernutrition, learning disorders, growth impairment, reduced productivity, and earning capacity in adulthood [6–8]. Focusing on Bolivia, although several studies have been conducted on the prevalence of intestinal parasitic infections by helminths and protozoa in rural communities [9–11], comprehensive studies on polyparasitic infections, anemia, and nutritional status have not been performed. Therefore, these types of studies can serve as baseline data for helminth and protozoal infections prevention and efficient geographical targeting of control effort strategies. Central to this research is to determine the prevalence of infections with helminths, protozoa, and risk factors associated with growth impairment and anemia in regions with polyparasitic (two or more parasite species) infections. Herein, we report our findings from different rural regions of Bolivia, the highlands and lowlands, known to be endemic to gastrointestinal parasite infections.

## 2. Materials and Methods

### 2.1. Study Areas and Population

Cross-sectional data were collected from schoolchildren in the Bolivian highlands and lowlands. The study sites in these different geographical regions are known to be endemic for helminths and protozoan gastrointestinal parasites. In the altiplano, surveys were conducted in different school units situated at an altitude between 3,810 and 4,050 meters above sea level (masl), with a permanent cold climate (7.9°C mean yearly temperature) and dry vegetation. Health and basic service conditions are regular to inappropriate; 55% have a piped water supply, barely 30% have a flush toilet or a latrine, and most (99%) of the households lack piped sewers. School units in the lowlands are situated in the region of Yungas, at an altitude of 600–980 masl. This region has a semitropical climate (23°C mean yearly temperature) with exuberant foliage and vegetation. Health conditions are better than in the highlands; 60% of the population has a piped water supply, 60% have flush toilets or latrines, and 33% have sanitary sewers.

The study population consisted of schoolchildren aged 5–13 years. A total of 790 children participated in the study, 229 and 561 from the highlands and lowlands, respectively.

### 2.2. Stool Collection and Analysis

The day before school visits, eligible subjects were provided with a clean, sterile stool container. The following morning, stool samples were collected, taken to the laboratory, and analyzed microscopically. All stool samples under high suspicion for amebic dysentery (i.e., bloody diarrhea with mucus) were carefully examined in fresh and in smears fixed and stained with Wright. A portion of these stool samples was also analyzed by microscopy using Ritchie´s formol ether concentration after Wright staining. Individuals were considered positive for a species of gastrointestinal parasite if the diagnostic stages (eggs, larva, or cysts) were observed in the sample during microscopic observation. A child was considered to have a polyparasitic infection if they were found to be positive for two or more than two of any species.

### 2.3. Hemoglobin Determination

Venous whole blood was collected and hemoglobin levels were measured using an HemoCue analyzer (Ångelholm, Sweden). According to the World Health Organization, mild, moderate, and severe anemia correspond to hemoglobin concentrations of 11–11.4 g/dL, 8–10.9 g/dL, and <8 g/dL in the lowlands, while hemoglobin cut-offs of 14.5–14.9 g/dL, 11.5–14.4 g/dL, and <11.5 g/dL define mild, moderate, and severe anemia, respectively, in the highlands [12].

### 2.4. Nutritional Assessment

Anthropometry is the most simple and reliable method for characterizing nutritional status. Anthropometric measurements, including height (cm) and weight (kg), were collected to determine the growth of children. Measurements were taken by trained local nurses after standardized procedures and regular calibration of equipment. Children were measured barefoot and in light clothing; height was measured with a stable mobile stadiometer (model 217; SECA, Hamburg, DE), rounding measurements to the nearest 1 mm. Weight was determined using a mechanical floor scale (model 750, SECA, Hamburg, DE) and recorded to the nearest 1 kg. Height for age (HAZ) and body mass index for age (BAZ) Z-scores were calculated using the WHO AnthroPlus software for children aged 5–19 years (version 1.0.4), relative to WHO reference standards for the year 2007 [13]. According to the WHO classification, children with a HAZ score of 2 or more standard deviation (SD) below average (HAZ score = ≤−2 SD) were considered stunted, while children who are wasted have a BAZ score of 2 or more SD below average (BAZ score = ≤−2 SD).

### 2.5. Statistical Methods

Categorical variables were described as absolute and relative frequencies, while continuous ones were described as the median (inter quartile range (IQr)). The inferential analyses for categorical and continuous covariates were performed by Fisher's exact test and the Mann-Whitney and Kruskal–Wallis ones, respectively. Three different and independent univariate and multivariate binary logistic regression model series were estimated, with as the outcome (dependent variable) the occurrence of moderate/severe anemia, moderately/severely stunting, and moderately/severely wasted. In all these models, five determinants were tested as independent risk factors: age class (11–13 vs 8–10 vs 5–7 years), sex (male vs female), location (highlands vs lowlands), helminth infection (any vs none), and protozoal infection (any vs none). All *p* values were obtained by the two-sided exact method at the conventional 5% significance level. Data were analyzed by R 4.1.1 (R Foundation for Statistical Computing, Vienna, Austria-https://www.r-project.org/).

## 3. Results

### 3.1. Population and Parasite Infections

The demographic structure of the study population and the prevalence of parasites infecting children are detailed in Table 1. A total of 790 children were surveyed for intestinal helminth and protozoan infections, 229 in the highlands and 561 in the lowlands. In terms of sex, similar percentages of females and males were observed in the two regions. Across all three age groups, significant differences in prevalence were observed between the highland and lowland regions. Over 60% and 20% of children were infected with protozoan and helminth parasites, respectively. The prevalence of helminth infection by *Hymenolepis nana* was significantly higher in the highlands than in the lowlands. Nonsignificant higher prevalences of pathogenic *Ascaris lumbricoides* and *Giardia lamblia* infections were found in the highlands as compared to lowlands, while Hookworm, *Trichuris trichiura, Strongyloides stercoralis,* and *Enterobius vermicularis* infections were only detected in the lowlands. Overall, significantly higher prevalences were mostly determined within the protozoa in the highlands, except for the higher prevalence of *Endolimax nana* infections in the lowlands. The predominance of *Entamoeba coli*, *Blastocystis hominis, Iodamoeba butschli, Chilomastix mesnili, and Entamoeba histolytica* parasites was higher in the highlands. Microscopic identification of *E. histolytica* hematophagous trophozoites was accomplished using fresh and fixed stool samples. As previously reported, hematophagy is considered a discriminative microscopic criterion to distinguish *E. histolytica* from *E. dispar* infection [14–16]. Over 70% of children from the highlands and 60% of children from the lowlands were coinfected with helminth and protozoan parasites.

### 3.2. Polyparasitic Infections and Parasite Prevalence

**Figure 1:**
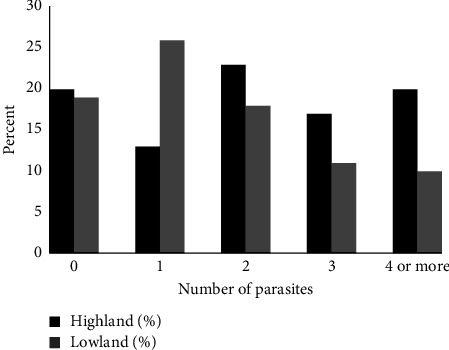
Percentage of children of highland and lowland regions with single and polyparasitic infections.

The percentage of children infected with one, two, three, or four or more parasites in the two regions is shown in Figure 1. Single parasitic infections were more prevalent in the lowlands, while higher percentages of polyparasitic infections, either of light (two parasites) or heavy (three or more parasites) intensity, were detected in the highlands. Overall, polyparasitic infections were observed in 78.6% and 66.7% of children from the highlands and lowlands, respectively (Table 1).

**Figure 2:**
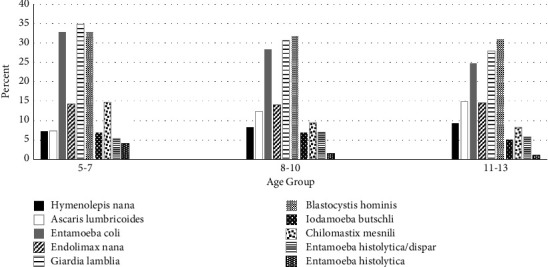
Parasite prevalence in children of highland and lowland regions according to age groups.

Figure 2 shows the age distribution of parasite prevalence. *E*. *coli*, *E. nana*, *G. lamblia*, and *B. hominis* were the most common protozoan infections in all age groups. Helminth infections by *H. nana* and *A. lumbricoides* predominated among older age children, particularly the 11- to 13-year-old group.

The prevalence of anemia and the analysis of anthropometric data are summarized in Table 2. Anemia was assessed in 771 children, 224 from the highlands and 547 from the lowlands. From the total number of children surveyed, 22.8% were mildly anemic, 43.7% anemic, and 1.8% severely anemic in the highlands, while 2.5% were mildly anemic, 1.6% anemic, and none of the children were severely anemic in the lowlands. Anthropometric data for HAZ and BAZ was obtained from 789 children, 229 and 560 were from highlands and lowlands, respectively. Overall, the nutritional status of the majority of children was within normal ranges, with a low prevalence of acute (BAZ ≤ −2 and ≤ −3) and chronic (HAZ ≤ −2 and ≤−3) undernutrition, respectively. Similar patterns of mildly stunting, stunting, and severe stunting were observed in both regions. A higher prevalence of marginally wasted (10% versus 4.1%) and wasted (2.6% versus 0.3%) children was observed in the highlands compared to the lowlands. Mildly stunting and marginally wasting were defined as < −1 *Z*-score.

Three different and independent series of uni- and multivariate binary logistic regression models have been estimated to determine the occurrence of moderate/severe anemia, moderate/severe stunting (HAZ), and moderate/severe wasting (BAZ); in all these series, we investigated the age class, sex, location, helminth, and protozoan-associated risk factors.

As for the first outcome, the ultimate determining factor associated with moderate/severe anemia was the highland location, which strongly increased the risk by around 50 times (OR 50.73, *p* < 0.001). In the univariate model, older ages (>8 years) were significant correlates of anemia (*p* < 0.001 and 0.007); however, these associations were no longer significant after adjustment for the other cofactors in the multivariate model (*p* = 0.504 and 0.643). Children's sex and concurrent infections by helminths and/or protozoa played no role. Results are summarized in Table 3.

Concerning moderate/severe stunting, only children with increasing ages emerged as a HAZ predictor. While the 8 to 10-year-old children showed no major risk when compared to the 5 to 7-year-old children (OR 1.29, *p* = 0.525), the 11 to 13-year-old class got more than double the elevated HAZ risk when compared to the youngest one (OR 2.28, *p* = 0.045) (Table 4).

Multivariate analysis indicated a highly significant association between highland location and wasting (OR 3.42, *p* = 0.025). The increased age of children (8–10 years) was likely a risk factor associated with wasting. However, this risk factor was marginally significant in a multivariate model (*p* = 0.065). Similarly, multivariate logistic regression modeling indicated a marginally significant association between male sex and wasting (*p* = 0.061). Concurrent helminth and/or protozoa infections, as in an altered BAZ, played no role.  Table 5 summarizes the findings.

## 4. Discussion

The findings of the present survey conducted in the highland and lowland regions of Bolivia showed that intestinal protozoa were predominant compared to helminths. The predominant pathogenic parasites in this study were *Giardia lamblia* and *Ascaris lumbricoides.* Overall, the high frequency of protozoan infections in the highlands is likely associated with the environmental setting and poor sanitary conditions, including contaminated water and latrine use. These findings highlight a higher risk of infection in children living and growing up in these conditions. Furthermore, the significantly higher prevalence of *H. nana, I. butschli, and C. mesnili* infections in the highlands, all of them with a fecal-oral transmission mode, indicates ingestion of contaminated water or food.

Periodic deworming of children was carried out by administering mebendazole; however, while ensuring effective action against helminths, this drug does not act against protozoan infection. Not coincidentally, the frequency of protozoan infection in the highland and lowland regions was higher than the helminth infections in this study.

Results of our study show a worryingly high prevalence of anemia in children of highlands in a multivariate regression model. Anemia is a global public health problem affecting approximately one-third of the world population [17], with ensuing effects on the physical and cognitive development of schoolchildren, predisposing them to a higher frequency of morbidity [18–20]. The predisposing factors to anemia in the highlands are nutritional and parasitic. First, iron deficiency is the most common type of anemia, and the availability of iron-rich food is scarce in the highlands. Children´s diets consist mostly of carbohydrates, vegetables, and fruits, and they hardly ever eat meat. Therefore, a low intake of iron may be one of the causes of the anemia observed in this population. Another proposed and plausible mechanism is related to anemia of inflammation or anemia of chronic disease [21–23], caused by recurrent parasitic infections that spread through unsafe water and sanitation along with poor hygiene in the highlands. However, more research is needed in this regard to assess the effect of the pattern and intensity of polyparasitism detected in the highlands on anemia of inflammation.

The survey of schoolchildren showed a more than double-increasing risk of chronic undernutrition (stunting) in children of the 11–13 year age group when compared to children 5–7 years old, making the older age of early adolescence (i.e., 10–14 years) [24] an important risk factor for stunting. This is commonly seen as a chronic manifestation resulting from prolonged undernourishment and repetitive infections; therefore, stunting is prominent amongst older children compared to younger age groups [25–27]. Regarding pathogens, intestinal malabsorption is a potential cause of linear growth faltering in children infected with *A. lumbricoides* and *G. lamblia*. However, infections by these parasites were not significant cofactors for stunting in this study. In addition, recurrent exposure to enteric pathogens, despite their low intensity and diarrheal disease, would have a detrimental effect on children's growth [28, 29]. Together with a low prevalence of stunted and severely stunted children, it was observed that there was a high prevalence of mildly stunted children in the two locations. In another study, children with this characteristic presented higher concentrations of plasma insulin, elevated glycemia, increased insulin resistance, and diminished pancreatic production of insulin in comparison with individuals of the normal body mass index [30]. This study points to an impairment of physiological mechanisms that regulate energy conservation and fat accumulation, leading to obesity and the risk of diabetes in adult life. Whether these metabolic alterations hold in our work remains to be confirmed.

A significant risk of wasting was determined for children living in the highlands. However, very few of the children surveyed were wasted. This fact may suggest a minor influence of infection by intestinal helminths on the nutrition of children when they have a low burden of parasites, as observed in this study. Wasting is associated with helminthiasis, while antihelminthic drug administration has been shown to prevent this debilitating disease [31].

The limitations of our survey relate first to the fact that a single stool sample per child was analyzed which may underestimate the exact prevalence of parasites. Second, there is a lack of data on the intensity of helminth infections since health impacts are intensity-related.

In this study, there was no association between concurrent infections by helminths and/or protozoa and anemia. Even though causality cannot be inferred from our cross-sectional survey design, recurrent parasitic infections as a result of poor hygiene and sanitary conditions and a diet deficient in iron, characteristics commonly found in highland communities, are predisposing factors to hemoglobin status. Based on our findings and those of other studies [22, 32, 33] more research is needed to determine whether anemia of inflammation caused by long-lasting parasitic infections together with iron deficiency likely contributes to anemia in our setting. The high prevalence of anemia in children of the highlands remains a public health concern. Considering its impact on childhood cognitive performance and physical fitness, their main causes must be identified as part of the integrated management of this disease.

## 5. Conclusions

Our findings highlight some key challenges that need to be addressed to improve childhood health conditions. The control of parasitic infections can only be attained with effective antiparasitic treatment and improved hygienic conditions to stop recurrent infections. Some measures are already in place and need improvement (i.e., deworming) while others, like improving water and environmental sanitation, are largely absent from these sites.

## Figures and Tables

**Table 1:** Demographic characteristics of helminth and protozoa infections among children in the highland and lowland regions of Bolivia.

	Total (*N* = 790) (%)	Highland (*N* = 229) (%)	Lowland (*N* = 561) (%)	*p* ^*a*^
**Sex**				
Female	53.9	29.6	70.4	
Male	46.1	28.3	71.7	0.753
**Age**				
5–7	31.3	42.9	57.1	
8–10	44.2	21.8	78.2	
11–13	24.6	24.2	75.8	<0.001
Helmimths				
*Hymenolepis nana*	8.2	15.7	5.2	<0.001
*Ascaris lumbricoides*	11.4	41.9	28.5	0.806
*N. americanus/A. duodenale*	0.5	0	0.5	0.329
*Trichuris trichiura*	1.5	0	0.7	0.047
*Strongyloides stercoralis*	1.3	0	0.2	0.071
*Enterobius vermicularis*	0.4	0	0.9	0.561
Overall prevalence	22	24.9	20.9	0.220
Protozoa				
*Entamoeba coli*	28.9	48.9	16	<0.001
*Endolimax nana*	14.2	9.6	31.7	0.018
*Giardia lamblia*	31.3	30.1	11.2	0.673
*Blastocystis hominis*	31.9	40.2	28.5	0.002
*Iodamoeba butschli*	6.5	16.1	2.5	<0.001
*Chilomastix mesnili*	10.8	19.2	7.3	<0.001
*Entamoeba histolytica/dispar*	6.1	7.4	5.5	0.326
*Entamoeba histolytica*	2.2	5.7	0.7	<0.001
Overall prevalence	62	74.2	57.0	0.001
Coinfection				
Helminths-protozoa^‡^	70.1	78.6	66.7	0.001

*p* ^*a*^ stands for significant differences between the study sites by chi-square test. ^‡^Overall prevalence of polyparasitic infections by helminth and protozoa.

**Table 2:** Hematologic, HAZ, and BAZ anthropometric measurements in children of highland and lowland regions of Bolivia.

	Total	Highland (H)	Lowland (L)	H/L (%)
Hematology				
Normal	595	71 (11.9%)	524 (88.1%)	31.7/95.7
Mildly anemic	65	51 (78.5%)	14 (21.5%)	22.8/2.5
Moderately anemic	107	98 (91.6%)	9 (8.4%)	43.7/1.6
Severely anemic	4	4 (100%)	0 (0%)	1.8/0
Missing	19			
TOTAL for hematology	790	224	547	
HAZ				
Normal	570	166 (29.1%)	404 (70.9%)	72.5/72.1
Mildly stunted	174	52 (29.9%)	122 (70.1%)	22.7/21.8
Stunted (≤−2SD)	34	8 (23.5%)	26 (76.5%)	3.5/4.6
Severely stunted (≤−3SD)	11	3 (27.3%)	8 (72.7%)	1.3/1.4
Missing	1			
TOTAL for HAZ	790	229	560	
BAZ				
Normal	729	198 (27.2%)	531 (72.8%)	86.5/94.8
Marginally wasted	46	23 (50.0%)	23 (50.0%)	10.0/4.1
Wasted (≤−2SD)	8	6 (75.0%)	2 (25.0%)	2.6/0.4
Severely wasted (≤−3SD)	6	2 (33.3%)	4 (66.7%)	0.9/0.7
Missing	1			
TOTAL for BAZ	790	229	560	

All percentages were calculated on rows after missing exclusion.

**Table 3:** Univariate and multivariate logistic regression models for moderate/severe anemia occurrence according to age, sex, location, and parasite infections.

	Univariate models			Multivariate model		
	OR	95% CI	*p*	OR	95% CI	*p*
Age (yrs)			<0.001			0.782
(8–10) vs (5–7)	0.41	0.26–0.65	<0.001	0.82	0.47–1.46	0.504
(11–13) vs (5–7)	0.48	0.28–0.82	0.007	0.86	0.45–1.64	0.643
Sex (M vs F)	1.11	0.74–1.66	0.613	—	—	—
Location (highland vs lowland)	49.98	24.56–101.59	<0.001	50.73	24.25–105.37	<0.001
Helminths (pos vs neg)	1.07	0.66–1.72	0.788	—	—	—
Protozoa (pos vs neg)	1.51	0.98–2.33	0.065	0.78	0.44–1.36	0.376

**Table 4:** Univariate and multivariate logistic regression models for moderate/severe stunting (HAZ) occurrence according to age, sex, location, and parasite infections.

	Univariate model			Multivariate model		
	OR	95% CI	*p*	OR	95% CI	*p*
Age (yrs)			0.098			0.098
(8–10) vs (5–7)	1.29	0.58–2.85	0.525	1.29	0.58–2.85	0.525
(11–13) vs (5–7)	2.28	1.02–5.09	0.045	2.28	1.02–5.09	0.045
Sex (M vs F)	0.94	0.51–1.71	0.828	—	—	—
Location (highland vs lowland)	0.78	0.39–1.57	0.487	—	—	—
Helminths (pos vs neg)	1.65	0.86–3.17	0.135	—	—	—
Protozoa (pos vs neg)	0.91	0.49–1.68	0.765	—	—	—

**Table 5:** Univariate and multivariate logistic regression models for moderately/severely wasted (BAZ) occurrence according to age, sex, location, and parasite infections.

	Univariate models			Multivariate model		
	OR	95% CI	*p*	OR	95% CI	*p*
Age (yrs)			0.045			0.118
(8–10) vs (5–7)	0.23	0.06–0.86	0.029	0.28	0.07–1.08	0.065
(11–13) vs (5–7)	0.28	0.06–1.29	0.102	0.34	0.07–1.63	0.177
Sex (M vs F)	2.99	0.93–9.61	0.066	3.06	0.95–9.89	0.061
Location (highland vs lowland)	3.34	1.14–9.74	0.027	3.42	1.17–10.00	0.025
Helminths (pos vs neg)	0.96	0.27–3.49	0.955	—	—	—
Protozoa (pos vs neg)	1.10	0.37–3.31	0.865	—	—	—

## Data Availability

The data that support the findings of this study are available within the article.
